# Inflammation and Organ Injury the Role of Substance P and Its Receptors

**DOI:** 10.3390/ijms24076140

**Published:** 2023-03-24

**Authors:** Zhixing Zhu, Madhav Bhatia

**Affiliations:** Department of Pathology and Biomedical Science, University of Otago, Christchurch 8140, New Zealand

**Keywords:** substance P, NK1R, MRGPRX2/B2, inflammation, organ injury

## Abstract

Tightly controlled inflammation is an indispensable mechanism in the maintenance of cellular and organismal homeostasis in living organisms. However, aberrant inflammation is detrimental and has been suggested as a key contributor to organ injury with different etiologies. Substance P (SP) is a neuropeptide with a robust effect on inflammation. The proinflammatory effects of SP are achieved by activating its functional receptors, namely the neurokinin 1 receptor (NK1R) receptor and mas-related G protein-coupled receptors X member 2 (MRGPRX2) and its murine homolog MRGPRB2. Upon activation, the receptors further signal to several cellular signaling pathways involved in the onset, development, and progression of inflammation. Therefore, excessive SP–NK1R or SP–MRGPRX2/B2 signals have been implicated in the pathogenesis of inflammation-associated organ injury. In this review, we summarize our current knowledge of SP and its receptors and the emerging roles of the SP–NK1R system and the SP–MRGPRX2/B2 system in inflammation and injury in multiple organs resulting from different pathologies. We also briefly discuss the prospect of developing a therapeutic strategy for inflammatory organ injury by disrupting the proinflammatory actions of SP via pharmacological intervention.

## 1. Introduction

Inflammation is a natural process activated by the immune system when the host organism is facing threats from infections and organ injuries of different etiologies [1]. Indeed, inflammation with rapid onset and acute resolution is indispensable in reestablishing cellular and organismal homeostasis disturbed by harmful stimuli [2]. Thus, a proper, effective, and tightly organized host inflammatory response acts as a protective biological process that enables us to survive under detrimental conditions [3]. By contrast, aberrant, nonresolving, and chronic inflammation are the leading contributors to multiple systemic diseases [4,5]. Notably, uncontrolled inflammation has been linked to the pathogenesis of many types of organ injuries, such as an acute lung injury attributed to sepsis, acute pancreatitis, burn injury, infections, and cigarette smoking exposure, as well as acute liver injury resulting from multiple etiologies, including sepsis, SARS-CoV-2 infection, and ischemia–reperfusion injury [6,7,8,9,10,11,12].

Substance P (SP, with an amino acid sequence of Arg-Pro-Lys-Pro-Gln-Gln-Phe-Phe-Gly-Leu-Met.NH_2_) was first discovered in 1931 by Von Euler and Gaddum [13]. Thereafter, many investigations have led to our current understanding that SP functions as an important mediator involved in a wide array of biological processes, including pain signaling, inflammation regulation, host defense response, and wound healing processes [14,15,16,17]. After its release, SP binds to, and subsequently primes, its functional receptors on the surface of effector cells, through which SP signals to several cellular signaling pathways and, consequently, exerts its multifaceted (patho)physiological functions [18,19].

Of note, is the recognition that inflammation is of great importance in organ injury [4] and that SP shapes the progression of inflammation [15], which prompts the extensive investigation of the actions played by SP in inflammation-associated organ injuries. For example, SP has been shown to be detrimental to several diseases related to acute inflammation, including sepsis/endotoxemia, acute pancreatitis, and burn injuries [20,21,22]. Likewise, the actions played by SP in chronic inflammation-associated disorders, including rheumatoid arthritis, chronic pruritus, and cancers have also been widely investigated [23,24,25].

Although several efforts have been made to elucidate the potential relationship between SP and inflammatory organ injury, the exact mechanism through which SP affects the pathophysiology of inflammation-associated organ injuries is not yet fully understood. In this review, we aim to provide an overview of the universal features of SP and its receptors. We also intend to summarize the present-day knowledge on the roles and corresponding mechanisms of SP and its receptors in inflammation-associated organ injuries. Finally, we discuss the prospect of developing a therapeutic strategy for inflammatory organ injury by disrupting the system of SP and its receptors.

## 2. Overview of Substance P and Its Receptors

SP has been implied in diverse biological processes and pathological settings. SP is not able to cross cellular membranes; thus, the functions of SP are mediated by its receptors. Briefly, the coupling of SP and its receptors triggers the activation of several secondary messengers. Upon priming, these downstream effectors further signal to multiple cellular signaling pathways, and, as a consequence, SP exerts its multifaceted functions in living organisms.

### 2.1. Overview of Substance P

**Figure 1:**
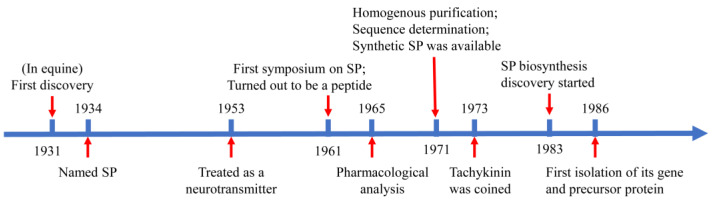
Historical timeline of substance P.

As illustrated in Figure 1, SP was originally isolated from tissue extracts of the equine intestine and brain tissues. Further, SP is able to induce muscle contractions and hypotension. More importantly, this atropine-resistant effect of SP is distinctive from that of choline and acetylcholine [13]. This substance was named SP because P stands for powder [13]. As elucidated, 30 years after its discovery, the nature of SP was first identified and it turned out that SP is a peptide. Significantly, SP was later found to be highly conserved among different mammalian species, with homologs in bovines, rodents, rabbits, and humans being gradually discovered. Excitingly, in 1971, SP was homogeneously purified. Subsequently, with the determination of the composition and sequence of amino acids comprising SP, it was found to be an undecapeptide, with a net positive charge at a physiological pH environment [26]. SP is probably the best-known member of the mammalian tachykinin neuropeptides family, which also comprises several structurally related neuropeptides, such as neurokinin A (NKA, with an amino acid sequence of His-Lys-Thr-Asp-Ser-Phe-Val-Gly-Leu-Met.NH_2_), neurokinin B (NKB, with an amino acid sequence of Asp-Met-His-Asp-Phe-Phe-Val-Gly-Leu-Met.NH_2_), neuropeptide K (NPK), and neuropeptide γ (NPγ) [27]. NPK and NPγ are two N-terminally extended forms of NKA, while NPγ lacks the residues 3–17 found in NPK.

**Figure 2:**
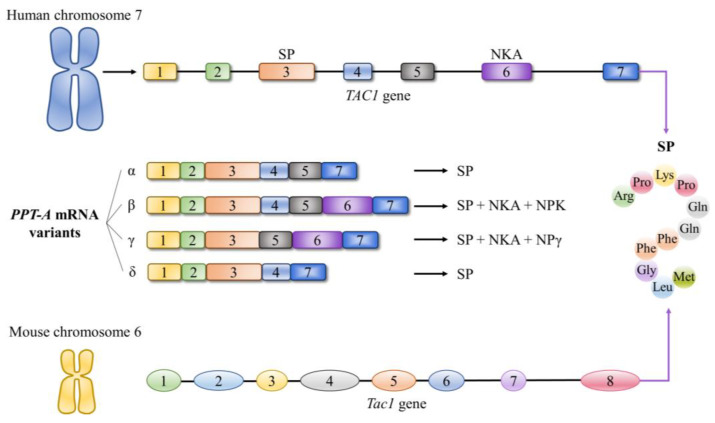
Overview of the characteristics of the corresponding gene encoding substance P (SP) in humans and mice. Different variants of the preprotachykinin-A (PPT-A) mRNA encode four different neuropeptides, including SP, neurokinin A (NKA), neuropeptide K (NPK), and neuropeptide γ (NPγ).

SP is a cleavage product of the precursor peptide preprotachykinin A. In humans, this prepeptide is encoded by the *TAC*1 gene, which is also called the preprotachykinin-A (*PPT-A*) gene and is located on chromosome 7. The *TAC*1 gene consists of seven exons and six introns, which can be alternatively spliced into four distinctive mRNA variants: *α*-*PPT-A* mRNA, *β*-*PPT-A* mRNA, *γ*-*PPT-A* mRNA, and *δ*-*PPT-A* mRNA [27]. As a consequence, the *TAC*1 gene is capable of encoding NKA, neuropeptide K, and neuropeptide γ, in addition to SP [28]. Notably, the gene that encodes mouse SP is the *Tac*1 (*Ppt-a*) gene. In contrast to the *TAC*1 gene, the *Tac*1 gene is located on chromosome 6 and comprises eight exons and seven introns (Figure 2).

As a biologically active peptide, SP is almost ubiquitously distributed throughout the body [28]. Since SP is categorized as a neuropeptide, it is predominantly detected in the central and peripheral nervous systems. Importantly, in addition to the cells located in the nervous system, such as neurons, astrocytes, microglia, epithelial cells, and endothelial cells [29,30,31], various other cells, especially the immune cells, including T cells, leukocytes, monocytes, macrophages, dendritic cells, lymphocytes, and eosinophils, have also been shown to produce and release significant amounts of SP [30]. Moreover, SP has been observed in some stem cells, including dental pulp stem cells and umbilical cord blood stem cells [32,33]. The widespread bioavailability of SP suggests that it could be extensively involved in a diverse range of (patho)physiological processes.

The structure of SP is also associated with the potential effects of SP, whereby it comprises 11 amino acids and has an amidation at the C-terminus. The building blocks of SP consist of two positively charged and six nonpolar amino acid residues [26]. Previous research has shown that the N-terminal segment of SP is crucial for the binding of SP to its receptors [34,35]. Specifically, most positively charged residues (polar) are located on the N-terminus, whereas the C-terminus contains more hydrophobic residues (uncharged). This spatial distribution of these residues confers amphiphilic properties to SP and, thus, allows SP to interact with the lipid bilayer of cellular membranes.

The half-life of SP is quite short in tissues, ranging from seconds to minutes [17,36]. Once released, SP binds to its receptors and forms a ligand/receptor complex. This is followed by rapid cellular internalization of this complex into the corresponding effector cells. After exposure to an acidic intracellular environment, SP detaches from the complex and is degraded by proteolytic enzymes, including the endothelin-converting enzyme-1 [37]. Unbound SP also undergoes a degradation process by neprilysin (a cell-surface metalloendopeptidase) soon after exocytosis [15,38]. By contrast, plasma SP has a higher stability, and it has been reported that SP is stable for hours in plasma [39]. This suggests that, on one hand, SP can trigger a prompt effect, while on the other hand, it is necessary to improve its stability in solvents.

Several signaling pathways have been shown to participate in the endogenous generation of SP. Host-generated hydrogen sulfide has been demonstrated as an upstream mediator involved in the upregulation of SP biosynthesis. In a mouse model of polymicrobial sepsis, elevated hydrogen sulfide biosynthesis was found to increase the endogenous production of SP by enhancing the expression and activity of the transient receptor potential vanilloid type 1 [40]. In a rat model of chronic constriction injury, the activation of the Wnt/β-catenin signaling pathway induced by nociceptive input has been linked to the elevation of the generation and release of SP [41]. The nuclear factor kappa B (NF-κB) pathway also contributed to the biosynthesis of SP in several immune cells [42].

### 2.2. Overview of Substance P Receptors

As discussed above, SP is able to interact with cellular membranes in lipid bilayers, however, it is not capable of crossing these membranes. As a result, the receptors to SP play an indispensable role in mediating the multifaceted effects of SP. The best-known functional receptor type for SP is the neurokinin receptor [28,35,43]. Importantly, mas-related G protein-coupled receptors X member 2 (MRGPRX2) in humans and its murine homolog named MRGPRB2 have been recently identified as other significant receptors for SP [44]. All of these receptors belong to the class I family of the seven-transmembrane, G protein-coupled receptors (GPCRs), however, they also differ from each other. Specifically, these receptors not only possess a different affinity to SP, yet they also mediate the different actions played by SP.

#### 2.2.1. Neurokinin Receptors

The neurokinin receptors family of SP comprises three members, which are the neurokinin 1 receptor (NK1R), neurokinin 2 receptor (NK2R), and neurokinin 3 receptor (NK3R) [28,35,43]. These neurokinin receptors are commonly located on the surface of the same cell and are simultaneously exposed to SP, however, NK1R has the highest affinity for SP, followed by NK2R, and NK3R. Therefore, NK1R is designated as an SP-preferring neurokinin receptor. Likewise, NK2R and NK3R are considered NKA-preferring neurokinin receptors and NKB-preferring neurokinin receptors due to their highest affinity being for NKA and NKB, respectively [28].

Apart from its highest affinity for SP, the widespread expression and similar bioavailability (both in the nervous system and other systems) of SP also lead to the recognition that NK1R is the major functional receptor of SP [43]. NK1R is encoded by the *TACR*1 gene in humans [27,43]. The *TACR*1 gene is located on chromosome two and has five exons and four introns, which allows the interruption of the protein-coding sequences (different isoforms of NK1R) to occur [27]. The mouse *Tacr*1 gene, consisting of nine exons and eight introns, and the mouse *Tac*1 gene are located on the same chromosome (chromosome six) [27].

**Figure 3:**
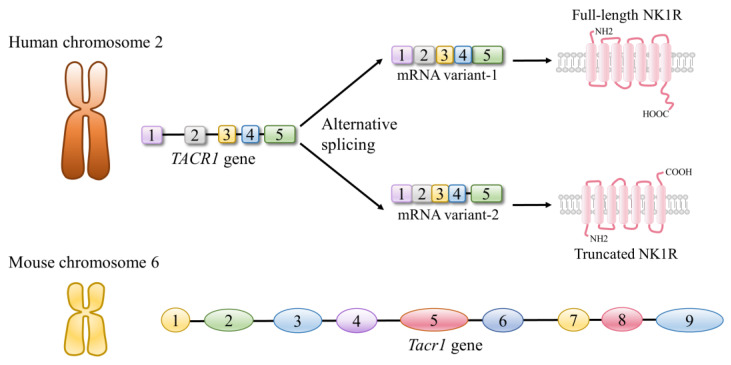
Overview of the characteristics of the corresponding gene encoding the neurokinin 1 receptor (NK1R) in humans and mice. The alternative splicing of the *TACR*1/*Tacr*1 gene leads to the presence of two variants of the *TACR*1/*Tacr*1 mRNA. These mRNA variants further translate to two NK1R isoforms, namely one full-length (with 407 amino acid residues) and one truncated (with 311 amino acid residues).

As shown in Figure 3, the resting NK1R is localized in the lipid rafts of cellular membranes, of whom, the microarchitecture and the composition affect the activation effect of SP on NK1R [45,46]. Once primed by SP, NK1R rapidly undergoes an endocytosis process (by endosomes) together with SP. The phosphate groups hydrolyzed from NK1R lead to an acidic intracellular environment surrounding the SP–NK1R complex into the cytoplasm. The acidification of the SP–NK1R complex further leads to the dissociation of this complex. While SP undergoes a degradation process, NK1R recycles to the cell surface, resulting in the de/re-sensitization process of NK1R [37]. In addition, NK1R can proceed to a process of ubiquitination and degradation, if it is under prolonged stimulation by SP [47].

**Figure 4:**
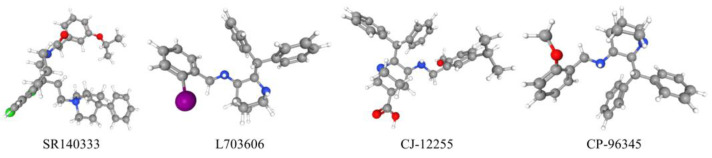
Three-dimensional structure of SR140333, L703606, CJ-12255, and CP-96345 (images were adapted from https://pubchem.ncbi.nlm.nih.gov/, accessed on 15 March 2023) [54].

There are two naturally occurring isoforms of NK1R. These two isoforms differ from each other in several aspects [48]. Firstly, the lengths of the encoded polypeptides that these two isoforms comprise are different. Specifically, the full-length one (NK1R-F), consists of 407 amino acid residues, while the truncated one (NK1R-T) only comprises 311 amino acid residues, as it is lacking 96 residues in the C-terminus [43,49,50]. In addition, NK1R-F and NK1R-T are differently distributed in the body. While NK1R-F is highly expressed at certain sites of the brain, including the striatum, caudate nucleus, putamen, globus pallidus, nucleus accumbent, and hypothalamus, NK1R-T is widely distributed throughout the body. As NK1R-F is the major form of NK1R in the brain, the expression of NK1R-T in the brain is relatively low [51]. Moreover, although the SP binding domain is identical in both NK1R isoforms, NK1R-F possesses an apparently higher binding affinity to SP than NK1R-T (more than 10-fold) [50]. Furthermore, the different compositions of the C-terminus result in distinctive functional property differences between NK1R-F and NK1R-T, as evidenced by the different effects of SP on the activation of extracellular signal-regulated kinases, such as protein kinase C-δ and NF-κB in HEK293 cells [52,53]. Apart from these, the lack of important amino acid residues in the C-terminus has also been linked to the altered de/re-sensitization and internalization processes of NK1R [43]. With the discovery and development of several effective antagonists targeting NK1R (Figure 4), including SR140333 (C_37_H_45_Cl_3_N_2_O_2_), L703606 (C_27_H_29_IN_2_), CJ-12255 (C_32_H_38_N_2_O_3_), and CP-96345 (C_28_H_32_N_2_O), the way that NK1R mediates the biological functions of SP has been widely investigated.

#### 2.2.2. Mas-Related G Protein-Coupled Receptors (MRGPRs)

MRGPRs refer to a family of CPCRs predominantly expressed in sensory neurons and extensively involved in relaying itchy and allergic signals [55,56]. Similarly, MRGPRs also belong to the class I family of GPCRs (δ-branch). Human MRGPRs comprise approximately 50 members and are further grouped into 9 subfamilies, which are categorized by their sequence similarities (MRGPRA-H and -X) [57]. Particularly, the subfamily X in the MRGPRs, designated as MRGPRXs, have been recognized as a group of primate-specific receptors (although not exclusively because their orthologs have been discovered in other species, including rodents) and have emerged as promising pharmacological targets in a broad range of diseases. There are four distinctive members in this subfamily, which are MRGPRX1, MRGPRX2, MRGPRX3, and MRGPRX4 [58].

Unlike the initial discoveries where the members of the MRGPRXs subfamily are only detected in dorsal root ganglia and trigeminal ganglia, recent evidence from several investigations has revealed that these receptors are also expressed in many other tissues [58]. MRGPRX2 shares the lowest degree of sequence identity and similarity with three other members in the MRGPRXs subfamily, however, it has the broadest distribution. MRGPRX2 and its murine ortholog MRGPRB2 have been detected in the skin, lungs, esophagus, and bladder, which contain mast cells. In addition to mast cells, MRGPRX2/B2 can be expressed by several other cell types, such as granulocytes, including blood basophils, eosinophils, and keratinocytes [59,60,61,62].

Of note, was the discovery that MRGPRX2/B2 is upregulated in inflammatory disorders and the recognition that MRGPRX2/B2 functions as a mast cell-specific receptor for SP (its affinity to SP is much lower than the canonical receptor of SP) have opened up a new era for MRGPRX2/B2 research [44,60]. MRGPRX2 consists of 330 amino acid residues and is encoded by the *MRGPRX*2 gene in humans. The gene is located on chromosome 11 and has 4 exons and 3 introns. In mice, MRGPRB2 is encoded by the *Mrgprb*2 gene, which is located on chromosome 7 and possesses 2 exons and 1 intron [58,60,63].

**Figure 5:**
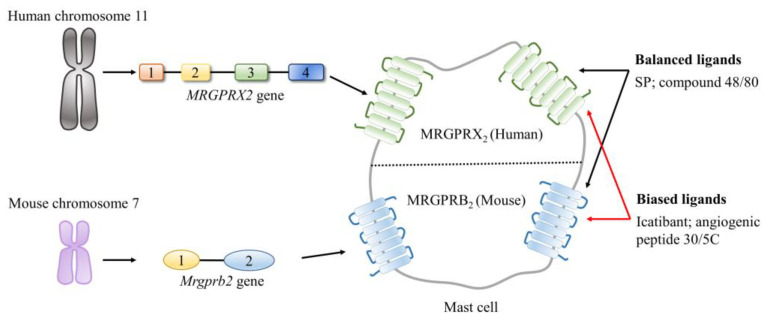
Overview of the characteristics of the corresponding gene encoding the mas-related G protein-coupled receptors member X2 (MRGPRX2)/B2 in humans and mice. MRGPRX2/B2 is a mast cell-specific receptor, with a broad range of ligands, including the balanced ligands (such a SP and compound 48/80) and the biased ligands (such as icatibant and angiogenic peptide 30/5C).

As illustrated in Figure 5, MRGCPRX2/B2 can be recognized and activated by a wide range of structurally and functionally diverse ligands. Specifically, these ligands either share little common sequence regularity, as peptide ligands or are categorized into different groups, ranging from small molecules to peptides/proteins. MRGPRX2/B2 behaves differently after its activation by different ligands [64]. On one hand, MRGPRX2/B2 rapidly undergoes an internalization and de/re-sensitization process once it is activated by balanced ligands (that induce both G-protein and β-arrestin signals, such as SP and a basic secretagogue named compound 48/80) [65,66]. On the other hand, primed MRGPRX2/B2 does not proceed to the internalization and de/re-sensitization process if it is activated by biased ligands (that only induce the G-protein signal, such as the angiogenic peptide-30/5C and icatibant) [67]. Moreover, in addition to the cellular surface of mast cells, MRGPRX2/B2 is present at the intracellular sites of mast cells [68]. These unique characteristics of MRGPRX2/B2 together distinguish it apart from other MRGPRXs.

## 3. Substance P and Its Receptors in Inflammation-Associated Organ Injury

The interplay between SP and its receptors results in the phosphorylation of several kinases, further leading to the activation or inactivation of many transcription factors, such as NF-κB, activator protein 1, and signal transducer and activator of transcription 6. As a result, the interaction of SP and NK1R or MRGPRX2/B2 can signal multiple pathways, including the mitogen-activated protein kinase (MAPK) pathway, the phosphoinositide 3-kinase (PI3K)-protein kinase B (PKB) pathway, and the NF-κB pathway in living organisms. Given that most of these pathways are extensively involved in the regulation of the generations of various cytokines and chemokines, as well as the recruitment and infiltration of immune cells, the SP–NK1R and SP–MRGPRX2/B2 systems are crucial to the modulation of inflammation progression and host defense response.

### 3.1. Emerging Roles of the SP–NK1R System in Inflammation-Associated Organ Injury

Emerging evidence has been highlighting the significant role of the SP–NK1R system in the pathogenesis of injury in multiple organs, including the lungs and liver, induced by inflammation under different conditions, such as sepsis, acute pancreatitis, and burn injuries [69].

#### 3.1.1. Role of the SP–NK1R System in Sepsis-Related Multiple Organ Injury

Sepsis is a life-threatening organ dysfunction. Sepsis occurs as a consequence of the failure of the host’s defense response to control invading pathogens and their toxins, which prompts the subsequent dysregulation of the immune response. Sepsis develops in around 30 million individuals worldwide and its incidence continues to rise and is responsible for approximately one-fifth of global deaths every year [40,70]. As indicated by its definition, sepsis is characterized by aberrant systematic inflammation and associated organ injury in response to a local infection. Increasing investigations have been pointing to the multifaceted actions played by the SP–NK1R system in sepsis-related inflammatory organ injuries, including lung injury, liver injury, and kidney injury [69,71].

In a landmark study conducted to explore the expression pattern of SP in mice with sepsis and to investigate the potential role of SP in sepsis-associated inflammation and acute lung injury, sepsis was established in mice by cecal ligation and puncture (CLP) [72]. In this study, the levels of SP were higher in mice with sepsis compared with control mice (both in the plasma and lungs). To explore the impact of SP on lung injury in sepsis, it was induced in genetically deficient *Tac*1-knockout mice. Importantly, the deletion of the *Tac*1 gene significantly attenuated the severity of inflammation and structural damage in lung tissues. Moreover, following sepsis, mice lacking the *Tac*1 gene had a better prognosis than wildtype mice, as evidenced by the delayed onset of lethality and higher survival probability. In addition, the deficiency of the *Tac*1 gene was linked to a lower level of systematic production of chemokines, recruitment of neutrophils, and bacterial burden, which underlies the detrimental effects of SP on lung inflammation and injury in sepsis, as well as the overall outcome of septic mice [72]. To further explore whether NK1R was involved in the proinflammatory effects of SP in sepsis-associated inflammation and lung injury, two highly potent and selective antagonists of NK1R, namely SR140333, and L703606, were used in a subsequent study [69]. In this study, the blockage of NK1R provided protection to mice against sepsis-induced inflammation in the lungs as the treatment of SR140333 or L703606 both led to a significant reduction in the levels of the production of chemokines and adhesion molecules alongside the subsequent infiltration of neutrophils and the release of proinflammatory cytokines in the lungs. In addition, NK1R blocking mitigated lung structural damage following sepsis. These results led to the conclusion that the detrimental effects of SP in sepsis-induced inflammation and lung injury are mediated by NK1R, indicating that NK1R antagonists could probably be of therapeutic benefit for sepsis [73]. Thereafter, it was demonstrated that by activating NK1R, SP led to the activation of the protein kinase C-α, thus, consequently, NK-κB and AP-1 were primed. The activation of NK-κB and AP-1 further caused inflammation and injury in the lungs from sepsis [74]. Furthermore, a microarray study demonstrated, for the first time, that the expression profile of genes involved in inflammation and immunomodulation in lungs were altered in the *Tac*1 gene-deficient mice, in comparison to the wildtype mice, shedding a more extensive insight into the proinflammatory impacts of SP on sepsis-induced acute lung injury [75]. The protective effects of NK1R blockage on cardiovascular function impaired in sepsis have also been demonstrated. It is reported that the treatment with CJ-12255, a specific antagonist for NK1R, significantly improved the chances of survival, pulse distension, and cardiac output in mice with sepsis. On the other hand, the blockage of NK1R also reduced proinflammatory cytokine and chemokine production and bacterial load [74]. Similarly, genetic deletion of the *Tacr*1 gene has been shown to result in the improvement of cardiovascular function, the decrease of inflammation, and the mortality of mice with CLP-induced sepsis [76]. More recently, it was reported that the deficiency of the *Tac*1 gene protected mice against sepsis-induced damage in the liver sinusoid, which may also underlie the detrimental impacts of SP on liver injury in CLP-induced sepsis [77]. In addition to sepsis, SP was also increased in endotoxemia caused by LPS injection in mice and contributed to the endotoxemia-induced injury in multiple organs, including the lungs, liver, and kidneys [78,79].

SP was also reported to be upregulated in septic patients. In a clinical investigation that recruited 61 patients with sepsis, which occurred after major visceral surgery, and 23 control cases, the plasma SP in sepsis patients was significantly higher than in control individuals [80]. More importantly, a higher level of plasma SP in the final phase of sepsis was linked to a worse outcome for sepsis patients in this study, as the level of plasma SP in 24 nonsurvivors was higher than in patients who survived [80]. Moreover, the levels of plasma SP have been found to be positively associated with the levels of proinflammatory mediators, such as procalcitonin, C-reactive protein, and interleukin-6, in patients with sepsis [21]. More recently, on top of its increase in septic patients, the response of SP to infections also varied with the site of infection, as the levels of SP in patients with abdominal infections were significantly higher than in patients with urinary tract infections [21].

Not surprisingly, several studies have also shown that SP underwent a decline in sepsis and a higher level of SP is probably beneficial to sepsis. Dating back to 1996, a clinical study showed that the level of SP in the plasma collected from patients with sepsis was significantly lower than that collected from healthy controls [80]. As SP is capable of inducing hypotension [13] and the blood pressure of septic patients tended to decrease [70], it was assumed that the decrease in plasma SP in septic patients was probably led by a compensatory mechanism trying to increase the blood pressure back to normal levels [80]. A research group has sequentially conducted two clinical investigations with a larger patient population and concluded that the 30-day survival proportion of septic patients with higher levels of plasma SP was higher than in patients with lower levels of plasma SP [81,82]. Noteworthy, the lack of healthy controls in these studies has led to the cross-sectional nature of these studies. Similarly, it was reported that disrupting the actions played by SP via the genetic deletion of the *Tacr*1 gene led to less efficacy in the elimination of bacteria, a higher level of inflammatory response, and a worse outcome in mice with staphylococcal sepsis [83].

#### 3.1.2. Roles of the SP–NK1R System in Acute Pancreatitis-Related Lung Injury

Acute pancreatitis, or acute inflammation of the pancreas, is a common pancreatic disorder and is characterized by a local and systemic inflammatory response [84]. The incidence of acute pancreatitis keeps increasing worldwide [85]. In addition to being a major cause of morbidity and mortality, acute pancreatitis is also a significant source of inflammatory organ dysfunction on a global scale [84,85]. The SP–NK1R system has been implicated in the pathogenesis of acute pancreatitis and associated inflammatory organ injury [69].

Back in 1997, it was reported that an intravascular injection of SP led to plasma extravasation in the pancreas by activating NK1R, suggesting that excessive SP–NK1R signaling could contribute to pancreatic injury [86]. The field has advanced significantly since a study that was conducted to explore the expression pattern and the potential role of the SP–NK1R system in acute pancreatitis [83]. In this study, acute pancreatitis was induced by intraperitoneal injection of caerulein. It was reported that caerulein injection significantly increased the expressions of SP and NK1R in the pancreas of wildtype mice [87]. Additionally, the pancreas tissue was severely damaged, as evidenced by higher levels of neutrophil infiltrating into the pancreas, acinar cell necrosis, and pancreatic edema in the pancreas tissues. Strikingly, the genetic deletion of the *Tacr*1 gene, disrupting the effects of SP, significantly mitigated caerulein-induced alterations in the pancreas. Similarly, mice with a deficiency in the *Tacr*1 gene were also protected against acute pancreatitis-related remote lung injuries, including neutrophils infiltration and elevated pulmonary microvascular permeability [87]. This research, for the first time, demonstrated an increased expression and proinflammatory effect of the SP–NK1R system in caerulein-induced acute pancreatitis and associated lung injury. To further elucidate the exact actions of SP in acute pancreatitis and associated lung injury, caerulein-induced acute pancreatitis was induced in wildtype mice and *Tac*1 gene-deficient mice [88]. Similar to the deletion of the *Tacr*1 gene, the deletion of the *Tac*1 gene was also shown to effectively mitigate caerulein-induced inflammatory injuries in the pancreas and lungs [88]. These findings prompted researchers to investigate whether it is possible to treat acute pancreatitis and associated lung injury by disrupting the SP–NK1R system with pharmacological interventions. To address this issue, CP-96345, a specific NK1R antagonist was used to explore the effects of pharmacological disruption to the SP–NK1R system for acute pancreatitis and associated lung injury in mice, as in their subsequent study [89]. The therapeutic effects of disrupting the SP–NK1R system were promising, as the treatment of CP-96345 effectively reduced the severity of caerulein-induced inflammatory injuries in the pancreas and lungs [89]. The proinflammatory effects of SP in acute pancreatitis and associated lung injury were further echoed by their subsequent research. In this study, the profile and role of neprilysin in acute pancreatitis and associated lung injury in mice were investigated [90]. It was reported that caerulein injection significantly inhibited the expression and activity of neprilysin. Since neprilysin catalyzes the degradation process of SP, the decline of neprilysin in its expression and activity resulting from the caerulein injection led to an obvious increase in SP production, which further caused inflammatory injuries to the pancreas and lungs in mice. In addition, inhibiting the activity of neprilysin, further, raised the production of SP and, consequently, exacerbated the inflammatory injuries in the pancreas and lungs [90].

A series of studies have been carried out to elucidate the underlying mechanisms through which the SP–NK1R system promotes caerulein-induced inflammatory injuries in the pancreas and lungs [91,92,93]. The excessive SP–NK1R signal enhanced the production and release of proinflammatory chemokines in multiple chemokine-secreting cells in the pancreas and lungs. As a result, caerulein-induced inflammatory injuries occur in the pancreas and lungs [91]. In addition to chemokines, the aberrant SP–NK1R system has been linked to a significant increase in the expressions of several adhesion molecules, which resulted in neutrophils accumulation and subsequent inflammatory injuries in the pancreas and lungs following caerulein injection [92]. Subsequently, the activation of several transcription factors, including STAT3, NF-κB, and AP-1 by the Src family kinases extracellular-signal-regulated kinase 1/2 (ERK1/2)/c-Jun NH(2)-terminal kinases pathway was found to participate in excessive SP–NK1R signal induced increase of adhesion molecules [93]. More recently, the SP–NK1R system was shown to activate the protein kinase C (PKC) α/MAPK pathway, which subsequently led to the increase of leukotriene B4 production and neutrophils reverse transendothelial cell migration. These alterations, ultimately, exaggerated the severity of acute pancreatitis and associated lung injury [94].

It is noteworthy that the protective effect of vitamin K3 in caerulein-induced acute pancreatitis and associated lung injury was partially attributed to its effect in inhibiting the NF-κB pathway and, consequently, decreased the production of SP and hydrogen sulfide [95]. Similarly, it has also been reported that the treatment of chaiqin chengqi decoction, a Chinese herbal formula commonly used to treat acute pancreatitis, protected mice against caerulein-induced inflammatory injury in the pancreas and lungs by inhibiting the SP–NK1R system [96]. The aforementioned beneficial effects of vitamin K3 and chaiqin chengqi decoction indirectly confirmed the proinflammatory actions of the SP–NK1R system in caerulein-induced acute pancreatitis and associated lung injury.

#### 3.1.3. Roles of the SP–NK1R System in Burn Injury Associated Lung Injury

Burn injury is a common pathological condition and is related to substantial morbidity and mortality [97]. The upregulation of multiple proinflammatory mediators alongside the onset, development, and progression of burn injuries, further, gives rise to an aberrant inflammation at the site of a burn injury [98]. Therefore, severe burn injuries can result in various disorders, such as sepsis, skeletal muscle dysfunction, and cognitive sequelae [98,99,100]. Accumulative evidence indicates that the SP–NK1R system is an important contributor to burn injury-induced inflammation and subsequent remote lung injury [69].

In a mouse model of burn injury, the 8 s period of immersion of the skin (30% total body surface area) in 95 °C water caused a burn injury. This burn injury led to an increase in the transcriptional levels of the genes coding for SP, NK1R, and proinflammatory mediators, including cytokines and chemokines in the lungs [101]. In addition, the levels of SP were also upregulated in the lungs and the elevated SP–NK1R expression correlated with lung inflammation and injury in mice after the induction of a burn injury on the skin [101]. Notably, the blockage of NK1R by the administration of an NK1R antagonist (L703606) resulted in significant protection for mice against burn injury-induced inflammation and injury in the lungs [101]. To further investigate the exact impacts of endogenous SP on burn injury-induced distant lung injury, a burn injury was induced, as mentioned above, in wildtype mice and mice lacking the *Tac*1 gene [102]. The induction of a burn injury significantly upregulated the production of SP alongside the increase in the inflammatory response and structural injury in the lungs of wildtype mice [102]. While the genetic deletion of the *Tac*1 gene mitigated burn injury-induced lung inflammation and injury, the supplementation of exogenous SP to the genetically deficient *Tac*1-knockout mice restored these burn injury-induced alterations in the lungs [102]. Importantly, with the analysis of the expression pattern of lung NK1R in mice subjected to a burn injury, it was concluded that the endogenous SP also contributed to the elevation in the expression of lung NK1R in mice subjected to burn injuries [102]. These results conclusively showed that SP contributes to systemic inflammation and lung injury following a burn injury. These aforementioned findings shed light on the detrimental effects of the SP–NK1R system in burn injury-associated lung inflammatory injuries. To further explore the downstream signaling pathways that mediate the proinflammatory roles of the SP–NK1R system in burn injury-related remote lung injury, mice were treated with specific inhibitors of ERK1/2 (PD98059) and NF-κB (BAY 11-7082) and exposed to a burn injury [22]. It was found that the local skin burn injury upregulated the SP–NK1R system, which further signaled to the ERK1/2-NF-κB pathway, thereby increasing the production of cyclooxygenase-2 and prostaglandin E metabolite and, ultimately, leading to remote lung inflammation and injury. The disruption of the SP–NK1R system, either by the genetic deletion of the *Tac*1 gene or the blockage of NK1R, abolished burn injury-induced activation of the ERK1/2-NF-κB pathway and protected mice from lung inflammation and injury. In addition, the treatment of the antagonists of ERK1/2 and NF-κB attenuated inflammation and injury in the lungs caused by excessive SP–NK1R signaling in this model of burn injury [22]. Likewise, in mice with smoke inhalation and burn injury, treatment with CP-96345 protected mice against inflammation and injury in the lungs [103].

### 3.2. Emerging Roles of the SP–MRGPRX2/B2 System in Inflammation-Associated Organ Injury

More recently, the SP–MRGPRX2/B2 axis has been implicated in the activation of mast cells of different origins. It was shown that the activation of MRGPRX2 by SP further signaled to the ERK1/2 pathway, thereby resulting in an IgE-independent activation of human umbilical cord blood-derived mast cells, as evidenced by increased degranulation and prostaglandin D2 (PGD2) release [104]. Similarly, it was reported that SP, acting on MRGPRX2/B2, triggered the production of histamine, 5-hydroxytryptamine, cytokines, and chemokines and degranulation in mast cells by activating ERK1/2, JNK, p38 MAPK, and PKB, as well as PKC and phospholipase C γ1 (involved in the calcium ion signaling pathway) [105,106,107]. Aberrant activation of mast cells has been implicated in multiple inflammatory disorders. Thus, excessive SP–MRGPRX2/B2 signal-mediated aberrant activation of mast cells has been regarded as a significant contributor to many inflammatory diseases, especially allergic disorders.

Chronic urticaria, a common allergic disease that affects approximately 1% of the global population, is characterized by the presence of skin wheals (hives), angioedema (swelling), or both [108,109,110]. The pathogenesis of chronic urticaria is complex. However, it is widely believed that the skin mast cells are the predominant effector cells in chronic urticaria [111]. Mast cell activation, degranulation, and release of histamines, PGD2, and tryptase, are critical processes in chronic urticaria [111]. Excessive SP–MRGPRX2 signaling has been implicated in the pathogenesis of chronic urticaria by the activation of skin mast cells [112]. Several studies have shown that the circulating levels of SP in patients with chronic urticaria were significantly higher than in the controls [113,114,115]. It has also been reported that the expression of MRGPRX2 is upregulated in skin-derived mast cells of patients with severe chronic urticaria [68]. Notably, the skin mast cells are shown to be activated by the treatment of SP, as evidenced by the increased release of histamine and PGD2, in a dose-dependent manner. Importantly, the effects of SP on the activation of skin mast cells have been shown to be achieved by priming MRGPRX2 rather than NK1R [68]. Excessive SP–MRGPRX2/B2 signaling has also been linked to the pathogenesis of several other allergic disorders, such as atopic dermatitis, asthma, and pseudoallergic reactions, as well as other pathological conditions, such as incision-induced inflammatory pain [19,44,116,117,118,119].

## 4. Conclusions

**Figure 6:**
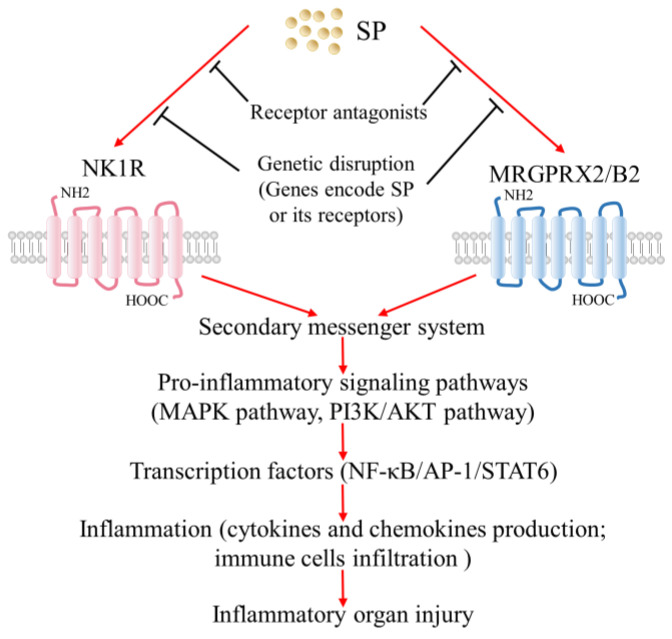
Schematic overview of the involvement of SP and its receptors in inflammatory organ injuries. Excessive SP–NK1R or SP–MRGPRX2/B2 signals lead to inflammatory organ injuries by promoting inflammation. This knowledge facilitates the development of novel treatments targeting the SP–NK1R system or the SP–MRGPRX2/B2 system for inflammatory organ injuries.

As a natural response raised by the host immune system in the face of threats, inflammation plays a central role in maintaining host homeostasis. However, aberrant inflammation tends to cause organ injury, thus, it is also treated as a significant contributor to many diseases. SP, a neuropeptide acting via its specific receptors NK1R or MRGPRX2/B2, promotes inflammation in multiple pathological conditions. Evidence has emerged that excessive SP–NK1R or SP–MRGPRX2/B2 signals are implicated in the pathogenesis of many inflammatory disorders and associated organ injuries, such as sepsis-associated lung/liver/kidney injury, acute pancreatitis-associated lung injury, burn injury-associated lung injury, and chronic urticaria-related skin alterations (Figure 6). Accumulating evidence in this area has prompted attempts to develop novel therapeutic approaches targeting the SP–NK1R system or the SP–MRGPRX2/B2 system for inflammatory organ injury, via pharmacological intervention. Although the mechanism of proinflammatory profile of SP and its receptors is not yet fully understood, it is worth putting more effort to explore the exact mechanism through which SP and its receptors participate in inflammatory organ injuries and to translate this knowledge into clinical practice in order to develop novel therapeutic approaches for several major health problems.

## Data Availability

Not applicable.

## References

[1] Oronsky B., Caroen S., Reid T. (2022). What Exactly Is Inflammation (and What Is It Not?). Int. J. Mol. Sci..

[2] Kotas M.E., Medzhitov R. (2015). Homeostasis, inflammation, and disease susceptibility. Cell.

[3] Netea M.G., Balkwill F., Chonchol M., Cominelli F., Donath M.Y., Giamarellos-Bourboulis E.J., Golenbock D., Gresnigt M.S., Heneka M.T., Hoffman H.M. (2017). A guiding map for inflammation. Nat. Immunol..

[4] Nathan C. (2022). Nonresolving inflammation redux. Immunity.

[5] Furman D., Campisi J., Verdin E., Carrera-Bastos P., Targ S., Franceschi C., Ferrucci L., Gilroy D.W., Fasano A., Miller G.W. (2019). Chronic inflammation in the etiology of disease across the life span. Nat. Med..

[6] Kumar V. (2020). Pulmonary Innate Immune Response Determines the Outcome of Inflammation During Pneumonia and Sepsis-Associated Acute Lung Injury. Front. Immunol..

[7] Zhu C.J., Yang W.G., Li D.J., Song Y.D., Chen S.Y., Wang Q.F., Liu Y.N., Zhang Y., Cheng B., Wu Z.W. (2021). Calycosin attenuates severe acute pancreatitis-associated acute lung injury by curtailing high mobility group box 1—Induced inflammation. World J. Gastroenterol..

[8] Comish P.B., Liu M.M., Huebinger R., Carlson D., Kang R., Tang D. (2022). The cGAS-STING pathway connects mitochondrial damage to inflammation in burn-induced acute lung injury in rat. Burns.

[9] Song Y., Miao S., Li Y., Fu H. (2019). Ulinastatin attenuates liver injury and inflammation in a cecal ligation and puncture induced sepsis mouse model. J. Cell Biochem..

[10] Choaib A., Issa E., El Choueiry F., Eldin J.N., Shbaklo K., Alhajj M., Sawaya R.T., Assi G., Nader M., Chatila R. (2022). SARS-CoV-2-mediated liver injury: Pathophysiology and mechanisms of disease. Inflamm. Res..

[11] Zhu Z., Lian X., Su X., Wu W., Zeng Y., Chen X. (2022). Exosomes derived from adipose-derived stem cells alleviate cigarette smoke-induced lung inflammation and injury by inhibiting alveolar macrophages pyroptosis. Respir. Res..

[12] Jiménez-Castro M.B., Cornide-Petronio M.E., Gracia-Sancho J., Peralta C. (2019). Inflammasome-Mediated Inflammation in Liver Ischemia-Reperfusion Injury. Cells.

[13] US V.E., Gaddum J.H. (1931). An unidentified depressor substance in certain tissue extracts. J. Physiol..

[14] Zieglgänsberger W. (2019). Substance P and pain chronicity. Cell Tissue Res..

[15] Suvas S. (2017). Role of Substance P Neuropeptide in Inflammation, Wound Healing, and Tissue Homeostasis. J. Immunol..

[16] Khorasani S., Boroumand N., Lavi Arab F., Hashemy S.I. (2020). The immunomodulatory effects of tachykinins and their receptors. J. Cell Biochem..

[17] Redkiewicz P. (2022). The Regenerative Potential of Substance P. Int. J. Mol. Sci..

[18] Ebrahimi S., Alalikhan A., Aghaee-Bakhtiari S.H., Hashemy S.I. (2022). The redox modulatory effects of SP/NK1R system: Implications for oxidative stress-associated disorders. Life Sci..

[19] Thapaliya M., Chompunud Na Ayudhya C., Amponnawarat A., Roy S., Ali H. (2021). Mast Cell-Specific MRGPRX2: A Key Modulator of Neuro-Immune Interaction in Allergic Diseases. Curr. Allergy Asthma Rep..

[20] Kumar A., Bhatia M. (2021). Role of Hydrogen Sulfide, Substance P and Adhesion Molecules in Acute Pancreatitis. Int. J. Mol. Sci..

[21] Gaddam R.R., Chambers S., Murdoch D., Shaw G., Bhatia M. (2017). Circulating levels of hydrogen sulfide and substance P in patients with sepsis. J. Infect..

[22] Sio S.W., Ang S.F., Lu J., Moochhala S., Bhatia M. (2010). Substance P upregulates cyclooxygenase-2 and prostaglandin E metabolite by activating ERK1/2 and NF-kappaB in a mouse model of burn-induced remote acute lung injury. J. Immunol..

[23] Ko K.R., Lee H., Han S.H., Ahn W., Kim D.K., Kim I.S., Jung B.S., Lee S. (2022). Substance P, A Promising Therapeutic Target in Musculoskeletal Disorders. Int. J. Mol. Sci..

[24] Coveñas R., Muñoz M. (2022). Involvement of the Substance P/Neurokinin-1 Receptor System in Cancer. Cancers.

[25] Ständer S., Yosipovitch G. (2019). Substance P and neurokinin 1 receptor are new targets for the treatment of chronic pruritus. Br. J. Dermatol..

[26] Chang M.M., Leeman S.E., Niall H.D. (1971). Amino-acid sequence of substance P. Nat. New Biol..

[27] Steinhoff M.S., von Mentzer B., Geppetti P., Pothoulakis C., Bunnett N.W. (2014). Tachykinins and their receptors: Contributions to physiological control and the mechanisms of disease. Physiol. Rev..

[28] Pennefather J.N., Lecci A., Candenas M.L., Patak E., Pinto F.M., Maggi C.A. (2004). Tachykinins and tachykinin receptors: A growing family. Life Sci..

[29] Navratilova E., Porreca F. (2019). Substance P and Inflammatory Pain: Getting It Wrong and Right Simultaneously. Neuron.

[30] Mashaghi A., Marmalidou A., Tehrani M., Grace P.M., Pothoulakis C., Dana R. (2016). Neuropeptide substance P and the immune response. Cell Mol. Life Sci..

[31] Kleczkowska P., Nowicka K., Bujalska-Zadrozny M., Hermans E. (2019). Neurokinin-1 receptor-based bivalent drugs in pain management: The journey to nowhere?. Pharmacol. Ther..

[32] Wei X.L., Luo L., Chen M.Z., Zhou J., Lan B.Y., Ma X.M., Chen W.X. (2022). Temporospatial Expression of Neuropeptide Substance P in Dental Pulp Stem Cells during Odontoblastic Differentiation in Vitro and Reparative Dentinogenesis in Vivo. J. Endod..

[33] Li Y., Douglas S.D., Ho W. (2000). Human stem cells express substance P gene and its receptor. J. Hematotherapy Stem Cell Res..

[34] Valentin-Hansen L., Park M., Huber T., Grunbeck A., Naganathan S., Schwartz T.W., Sakmar T.P. (2014). Mapping substance P binding sites on the neurokinin-1 receptor using genetic incorporation of a photoreactive amino acid. J. Biol. Chem..

[35] Harris J.A., Faust B., Gondin A.B., Dämgen M.A., Suomivuori C.M., Veldhuis N.A., Cheng Y., Dror R.O., Thal D.M., Manglik A. (2022). Selective G protein signaling driven by substance P-neurokinin receptor dynamics. Nat. Chem. Biol..

[36] Saidi M., Kamali S., Beaudry F. (2016). Characterization of Substance P processing in mouse spinal cord S9 fractions using high-resolution Quadrupole-Orbitrap mass spectrometry. Neuropeptides.

[37] Grady E.F., Garland A.M., Gamp P.D., Lovett M., Payan D.G., Bunnett N.W. (1995). Delineation of the endocytic pathway of substance P and its seven-transmembrane domain NK1 receptor. Mol. Biol. Cell.

[38] Sankhe R., Pai S.R.K., Kishore A. (2021). Tumour suppression through modulation of neprilysin signaling: A comprehensive review. Eur. J. Pharmacol..

[39] Rameshwar P., Joshi D.D., Yadav P., Qian J., Gascon P., Chang V.T., Anjaria D., Harrison J.S., Song X. (2001). Mimicry between neurokinin-1 and fibronectin may explain the transport and stability of increased substance P immunoreactivity in patients with bone marrow fibrosis. Blood.

[40] Zhu Z., Chambers S., Zeng Y., Bhatia M. (2022). Gases in Sepsis: Novel Mediators and Therapeutic Targets. Int. J. Mol. Sci..

[41] Li Y.S., Xi Y., Li X.J., Leng C.L., Jia M.M., Zhang W.K., Tang H.B. (2015). Up-Regulation of the Biosynthesis and Release of Substance P through Wnt/β-Catenin Signaling Pathway in Rat Dorsal Root Ganglion Cells. PLoS ONE.

[42] Blum A., Setiawan T., Hang L., Stoyanoff K., Weinstock J.V. (2008). Interleukin-12 (IL-12) and IL-23 induction of substance p synthesis in murine T cells and macrophages is subject to IL-10 and transforming growth factor beta regulation. Infect. Immun..

[43] Schank J.R., Heilig M. (2017). Substance P and the Neurokinin-1 Receptor: The New CRF. Int. Rev. Neurobiol..

[44] Green D.P., Limjunyawong N., Gour N., Pundir P., Dong X. (2019). A Mast-Cell-Specific Receptor Mediates Neurogenic Inflammation and Pain. Neuron.

[45] Monastyrskaya K., Hostettler A., Buergi S., Draeger A. (2005). The NK1 receptor localizes to the plasma membrane microdomains, and its activation is dependent on lipid raft integrity. J. Biol. Chem..

[46] Thom C., Ehrenmann J., Vacca S., Waltenspühl Y., Schöppe J., Medalia O., Plückthun A. (2021). Structures of neurokinin 1 receptor in complex with G(q) and G(s) proteins reveal substance P binding mode and unique activation features. Sci. Adv..

[47] Cottrell G.S., Padilla B., Pikios S., Roosterman D., Steinhoff M., Gehringer D., Grady E.F., Bunnett N.W. (2006). Ubiquitin-dependent down-regulation of the neurokinin-1 receptor. J. Biol. Chem..

[48] Spitsin S., Pappa V., Douglas S.D. (2018). Truncation of neurokinin-1 receptor-Negative regulation of substance P signaling. J. Leukoc. Biol..

[49] Lai J.P., Ho W.Z., Kilpatrick L.E., Wang X., Tuluc F., Korchak H.M., Douglas S.D. (2006). Full-length and truncated neurokinin-1 receptor expression and function during monocyte/macrophage differentiation. Proc. Natl. Acad. Sci. USA.

[50] Fong T.M., Anderson S.A., Yu H., Huang R.R., Strader C.D. (1992). Differential activation of intracellular effector by two isoforms of human neurokinin-1 receptor. Mol. Pharmacol..

[51] Caberlotto L., Hurd Y.L., Murdock P., Wahlin J.P., Melotto S., Corsi M., Carletti R. (2003). Neurokinin 1 receptor and relative abundance of the short and long isoforms in the human brain. Eur. J. Neurosci..

[52] Lai J.P., Lai S., Tuluc F., Tansky M.F., Kilpatrick L.E., Leeman S.E., Douglas S.D. (2008). Differences in the length of the carboxyl terminus mediate functional properties of neurokinin-1 receptor. Proc. Natl. Acad. Sci. USA.

[53] Chang C.T., Jiang B.Y., Chen C.C. (2019). Ion Channels Involved in Substance P-Mediated Nociception and Antinociception. Int. J. Mol. Sci..

[54] PubChem, Explore Chemistry Quickly Find Chemical Information from Authoritative Sources. https://pubchem.ncbi.nlm.nih.gov.

[55] Meixiong J., Dong X. (2017). Mas-Related G Protein-Coupled Receptors and the Biology of Itch Sensation. Annu. Rev. Genet..

[56] Yang F., Guo L., Li Y., Wang G., Wang J., Zhang C., Fang G.X., Chen X., Liu L., Yan X. (2021). Structure, function and pharmacology of human itch receptor complexes. Nature.

[57] Solinski H.J., Gudermann T., Breit A. (2014). Pharmacology and signaling of MAS-related G protein-coupled receptors. Pharmacol. Rev..

[58] Al Hamwi G., Riedel Y.K., Clemens S., Namasivayam V., Thimm D., Müller C.E. (2022). MAS-related G protein-coupled receptors X (MRGPRX): Orphan GPCRs with potential as targets for future drugs. Pharmacol. Ther..

[59] Roy S., Chompunud Na Ayudhya C., Thapaliya M., Deepak V., Ali H. (2021). Multifaceted MRGPRX2: New insight into the role of mast cells in health and disease. J. Allergy Clin. Immunol..

[60] Quan P.L., Sabaté-Brescó M., Guo Y., Martín M., Gastaminza G. (2021). The Multifaceted Mas-Related G Protein-Coupled Receptor Member X2 in Allergic Diseases and Beyond. Int. J. Mol. Sci..

[61] Wedi B., Gehring M., Kapp A. (2020). The pseudoallergen receptor MRGPRX2 on peripheral blood basophils and eosinophils: Expression and function. Allergy.

[62] Kiatsurayanon C., Niyonsaba F., Chieosilapatham P., Okumura K., Ikeda S., Ogawa H. (2016). Angiogenic peptide (AG)-30/5C activates human keratinocytes to produce cytokines/chemokines and to migrate and proliferate via MrgX receptors. J. Dermatol. Sci..

[63] Serhan N., Cenac N., Basso L., Gaudenzio N. (2021). Mas-related G protein-coupled receptors (Mrgprs)—Key regulators of neuroimmune interactions. Neurosci. Lett..

[64] Ogasawara H., Noguchi M. (2021). Therapeutic Potential of MRGPRX2 Inhibitors on Mast Cells. Cells.

[65] Chompunud Na Ayudhya C., Amponnawarat A., Ali H. (2021). Substance P Serves as a Balanced Agonist for MRGPRX2 and a Single Tyrosine Residue Is Required for β-Arrestin Recruitment and Receptor Internalization. Int. J. Mol. Sci..

[66] Lazki-Hagenbach P., Kleeblatt E., Ali H., Sagi-Eisenberg R. (2022). Spatiotemporal Patterns of Substance P-Bound MRGPRX2 Reveal a Novel Connection Between Macropinosome Resolution and Secretory Granule Regeneration in Mast Cells. Front. Immunol..

[67] Roy S., Ganguly A., Haque M., Ali H. (2019). Angiogenic Host Defense Peptide AG-30/5C and Bradykinin B(2) Receptor Antagonist Icatibant Are G Protein Biased Agonists for MRGPRX2 in Mast Cells. J. Immunol..

[68] Fujisawa D., Kashiwakura J., Kita H., Kikukawa Y., Fujitani Y., Sasaki-Sakamoto T., Kuroda K., Nunomura S., Hayama K., Terui T. (2014). Expression of Mas-related gene X2 on mast cells is upregulated in the skin of patients with severe chronic urticaria. J. Allergy Clin. Immunol..

[69] Bhatia M. (2015). H₂S and substance P in inflammation. Methods Enzym..

[70] Cecconi M., Evans L., Levy M., Rhodes A. (2018). Sepsis and septic shock. Lancet.

[71] Manandhar S., Sinha P., Ejiwale G., Bhatia M. (2021). Hydrogen Sulfide and its Interaction with Other Players in Inflammation. Adv. Exp. Med. Biol..

[72] Puneet P., Hegde A., Ng S.W., Lau H.Y., Lu J., Moochhala S.M., Bhatia M. (2006). Preprotachykinin-A gene products are key mediators of lung injury in polymicrobial sepsis. J. Immunol..

[73] Hegde A., Zhang H., Moochhala S.M., Bhatia M. (2007). Neurokinin-1 receptor antagonist treatment protects mice against lung injury in polymicrobial sepsis. J. Leukoc. Biol..

[74] Hegde A., Koh Y.H., Moochhala S.M., Bhatia M. (2010). Neurokinin-1 receptor antagonist treatment in polymicrobial sepsis: Molecular insights. Int. J. Inflam..

[75] Hegde A., Tamizhselvi R., Manikandan J., Melendez A.J., Moochhala S.M., Bhatia M. (2010). Substance P in polymicrobial sepsis: Molecular fingerprint of lung injury in preprotachykinin-A−/− mice. Mol. Med..

[76] Mella J.R., Stucchi A.F., Duffy E.R., Remick D.G. (2019). Neurokinin-1 Receptor Deficiency Improves Survival in Murine Polymicrobial Sepsis Through Multiple Mechanisms in Aged Mice. Shock.

[77] Gaddam R.R., Chambers S., Fraser R., Cogger V.C., Le Couteur D.G., Ishii I., Bhatia M. (2019). Cystathionine-Gamma-Lyase-Derived Hydrogen Sulfide-Regulated Substance P Modulates Liver Sieve Fenestrations in Caecal Ligation and Puncture-Induced Sepsis. Int. J. Mol. Sci..

[78] Ng S.W., Zhang H., Hegde A., Bhatia M. (2008). Role of preprotachykinin-A gene products on multiple organ injury in LPS-induced endotoxemia. J. Leukoc. Biol..

[79] Wang M., Zhong D., Dong P., Song Y. (2018). Blocking CXCR1/2 contributes to amelioration of lipopolysaccharide-induced sepsis by downregulating substance P. J. Cell Biochem..

[80] Arnalich F., Hernanz A., Jiménez M., López J., Tato E., Vázquez J.J., Montiel C. (1996). Relationship between circulating levels of calcitonin gene-related peptide, nitric oxide metabolites and hemodynamic changes in human septic shock. Regul. Pept..

[81] Lorente L., Martín M.M., Almeida T., Hernández M., Ferreres J., Solé-Violán J., Labarta L., Díaz C., Jiménez A. (2015). Association between serum substance P levels and mortality in patients with severe sepsis. J. Crit. Care.

[82] Lorente L., Martín M.M., Pérez-Cejas A., Ferreres J., Solé-Violán J., Labarta L., Díaz C., Jiménez A. (2017). Sustained Low Serum Substance P Levels in Non-Surviving Septic Patients. Int. J. Mol. Sci..

[83] Verdrengh M., Tarkowski A. (2008). The impact of substance P signalling on the development of experimental staphylococcal sepsis and arthritis. Scand. J. Immunol..

[84] Gardner T.B. (2021). Acute Pancreatitis. Ann. Intern. Med..

[85] Xiao A.Y., Tan M.L., Wu L.M., Asrani V.M., Windsor J.A., Yadav D., Petrov M.S. (2016). Global incidence and mortality of pancreatic diseases: A systematic review, meta-analysis, and meta-regression of population-based cohort studies. Lancet Gastroenterol. Hepatol..

[86] Figini M., Emanueli C., Grady E.F., Kirkwood K., Payan D.G., Ansel J., Gerard C., Geppetti P., Bunnett N. (1997). Substance P and bradykinin stimulate plasma extravasation in the mouse gastrointestinal tract and pancreas. Am. J. Physiol..

[87] Bhatia M., Saluja A.K., Hofbauer B., Frossard J.L., Lee H.S., Castagliuolo I., Wang C.C., Gerard N., Pothoulakis C., Steer M.L. (1998). Role of substance P and the neurokinin 1 receptor in acute pancreatitis and pancreatitis-associated lung injury. Proc. Natl. Acad. Sci. USA.

[88] Bhatia M., Slavin J., Cao Y., Basbaum A.I., Neoptolemos J.P. (2003). Preprotachykinin-A gene deletion protects mice against acute pancreatitis and associated lung injury. Am. J. Physiol. Gastrointest. Liver Physiol..

[89] Lau H.Y., Wong F.L., Bhatia M. (2005). A key role of neurokinin 1 receptors in acute pancreatitis and associated lung injury. Biochem. Biophys. Res. Commun..

[90] Koh Y.H., Moochhala S., Bhatia M. (2011). The role of neutral endopeptidase in caerulein-induced acute pancreatitis. J. Immunol..

[91] Sun J., Bhatia M. (2007). Blockade of neurokinin-1 receptor attenuates CC and CXC chemokine production in experimental acute pancreatitis and associated lung injury. Am. J. Physiology. Gastrointest. Liver Physiol..

[92] Lau H.Y., Bhatia M. (2007). Effect of CP-96,345 on the expression of adhesion molecules in acute pancreatitis in mice. Am. J. Physiology. Gastrointest. Liver Physiol..

[93] Ramnath R.D., Sun J., Bhatia M. (2009). Involvement of SRC family kinases in substance P-induced chemokine production in mouse pancreatic acinar cells and its significance in acute pancreatitis. J. Pharmacol. Exp. Ther..

[94] Li B., Han X., Ye X., Ni J., Wu J., Dai J., Wu Z., Chen C., Wan R., Wang X. (2018). Substance P-regulated leukotriene B4 production promotes acute pancreatitis-associated lung injury through neutrophil reverse migration. Int. Immunopharmacol..

[95] Amiti, Tamizhselvi R., Manickam V. (2019). Menadione (vitamin K3) inhibits hydrogen sulfide and substance P via NF-κB pathway in caerulein-induced acute pancreatitis and associated lung injury in mice. Pancreatology.

[96] Han C., Du D., Wen Y., Li J., Wang R., Jin T., Yang J., Shi N., Jiang K., Deng L. (2021). Chaiqin chengqi decoction ameliorates acute pancreatitis in mice via inhibition of neuron activation-mediated acinar cell SP/NK1R signaling pathways. J. Ethnopharmacol..

[97] Jeschke M.G., van Baar M.E., Choudhry M.A., Chung K.K., Gibran N.S., Logsetty S. (2020). Burn injury. Nat. Rev. Dis. Prim..

[98] Zhang P., Zou B., Liou Y.C., Huang C. (2021). The pathogenesis and diagnosis of sepsis post burn injury. Burn. Trauma.

[99] Knuth C.M., Auger C., Jeschke M.G. (2021). Burn-induced hypermetabolism and skeletal muscle dysfunction. Am. J. Physiol. Cell Physiol..

[100] Xie C., Hu J., Cheng Y., Yao Z. (2022). Researches on cognitive sequelae of burn injury: Current status and advances. Front. Neurosci..

[101] Sio S.W., Puthia M.K., Lu J., Moochhala S., Bhatia M. (2008). The neuropeptide substance P is a critical mediator of burn-induced acute lung injury. J. Immunol..

[102] Sio S.W., Moochhala S., Lu J., Bhatia M. (2010). Early protection from burn-induced acute lung injury by deletion of preprotachykinin-A gene. Am. J. Respir. Crit. Care Med..

[103] Jacob S., Deyo D.J., Cox R.A., Jacob R.K., Herndon D.N., Traber D.L., Hawkins H.K. (2010). Substance P antagonist CP-96345 blocks lung vascular leakage and inflammation more effectively than its stereoisomer CP-96344 in a mouse model of smoke inhalation and burn injury. Toxicol. Mech. Methods.

[104] Ogasawara H., Furuno M., Edamura K., Noguchi M. (2019). Novel MRGPRX2 antagonists inhibit IgE-independent activation of human umbilical cord blood-derived mast cells. J. Leukoc. Biol..

[105] Chaki S., Alkanfari I., Roy S., Amponnawarat A., Hui Y., Oskeritzian C.A., Ali H. (2021). Inhibition of Orai Channel Function Regulates Mas-Related G Protein-Coupled Receptor-Mediated Responses in Mast Cells. Front. Immunol..

[106] Che D., Zheng Y., Hou Y., Du X., Jia T., Zhao Q., Song X., Zhou T., Geng S. (2021). Action of substance P and PAMP(9–20) on different excitation sites of MRGPRX2 induces differences in mast cell activation. Int. Immunopharmacol..

[107] Hsin L., Fernandopulle N.A., Ding J., Lumb C., Veldhuis N., Karas J.A., Northfield S.E., Mackay G.A. (2022). The effect of substance P and its common in vivo-formed metabolites on MRGPRX2 and human mast cell activation. Pharmacol. Res. Perspect.

[108] Antia C., Baquerizo K., Korman A., Bernstein J.A., Alikhan A. (2018). Urticaria: A comprehensive review: Epidemiology, diagnosis, and work-up. J. Am. Acad. Dermatol..

[109] Gonçalo M., Gimenéz-Arnau A., Al-Ahmad M., Ben-Shoshan M., Bernstein J.A., Ensina L.F., Fomina D., Galvàn C.A., Godse K., Grattan C. (2021). The global burden of chronic urticaria for the patient and society. Br. J. Dermatol..

[110] Lang D.M. (2022). Chronic Urticaria. N. Engl. J. Med..

[111] Church M.K., Kolkhir P., Metz M., Maurer M. (2018). The role and relevance of mast cells in urticaria. Immunol. Rev..

[112] Vena G.A., Cassano N., Di Leo E., Calogiuri G.F., Nettis E. (2018). Focus on the role of substance P in chronic urticaria. Clin. Mol. Allergy.

[113] Metz M., Krull C., Hawro T., Saluja R., Groffik A., Stanger C., Staubach P., Maurer M. (2014). Substance P is upregulated in the serum of patients with chronic spontaneous urticaria. J. Investig. Dermatol..

[114] Zheng W., Wang J., Zhu W., Xu C., He S. (2016). Upregulated expression of substance P in basophils of the patients with chronic spontaneous urticaria: Induction of histamine release and basophil accumulation by substance P. Cell Biol. Toxicol..

[115] Fadaee J., Khoshkhui M., Emadzadeh M., Hashemy S.I., Farid Hosseini R., Jabbari Azad F., Ahanchian H., Lavi Arab F. (2020). Evaluation of Serum Substance P Level in Chronic Urticaria and Correlation with Disease Severity. Iran. J. Allergy Asthma Immunol..

[116] Kawakami Y., Yumoto K., Kawakami T. (2007). An improved mouse model of atopic dermatitis and suppression of skin lesions by an inhibitor of Tec family kinases. Allergol. Int..

[117] Serhan N., Basso L., Sibilano R., Petitfils C., Meixiong J., Bonnart C., Reber L.L., Marichal T., Starkl P., Cenac N. (2019). House dust mites activate nociceptor-mast cell clusters to drive type 2 skin inflammation. Nat. Immunol..

[118] Wang N., Wang J., Zhang Y., Zeng Y., Hu S., Bai H., Hou Y., Wang C., He H., He L. (2021). Imperatorin ameliorates mast cell-mediated allergic airway inflammation by inhibiting MRGPRX2 and CamKII/ERK signaling pathway. Biochem. Pharmacol..

[119] Xue Z., Zhang Y., Zeng Y., Hu S., Bai H., Wang J., Jing H., Wang N. (2021). Licochalcone A inhibits MAS-related GPR family member X2-induced pseudo-allergic reaction by suppressing nuclear migration of nuclear factor-κB. Phytother. Res..

